# ^82^Rb and [^15^O]H_2_O myocardial perfusion PET imaging: a prospective head to head comparison

**DOI:** 10.1007/s12350-023-03372-7

**Published:** 2023-10-03

**Authors:** Martin Krakauer, Afefah Ismail, Ulrik Talleruphuus, Alexander Cuculiza Henriksen, Markus N. Lonsdale, Inge Lise Rasmussen, Stefan Fuglsang, Eva Prescott, Peter Hovind, Lisbeth Marner

**Affiliations:** 1 Department of Clinical Physiology and Nuclear Medicine, Copenhagen University Hospital – Bispebjerg and Frederiksberg, Copenhagen, Denmark; 2 Department of Cardiology, Copenhagen University Hospital – Bispebjerg and Frederiksberg, Copenhagen, Denmark; 3 Department of Clinical Medicine, University of Copenhagen, Copenhagen, Denmark

**Keywords:** Cardiac imaging, myocardial perfusion, H_2_O, myocardial blood flow, extraction fraction, radiowater, positron emission tomography

## Abstract

**Background:**

^82^Rb PET and [^15^O]H_2_O PET are both validated tracers for myocardical perfusion imaging but have not previously been compared clinically. During our site’s transition from ^82^Rb to [^15^O]H_2_O PET, we performed a head-to-head comparison in a mixed population with suspected ischemic heart disease.

**Methods:**

A total of 37 patients referred for perfusion imaging due to suspicion of coronary stenosis were examined with both ^82^Rb and [^15^O]H_2_O PET on the same day in rest and during adenosine-induced stress. The exams were rated by two blinded readers as normal, regional ischemia, globally reduced myocardial perfusion, or myocardial scarring. For [^15^O]H_2_O PET, regional ischemia was defined as two neighboring segments with average stress perfusion ≤ 2.3 mL/(min·g). Further, we evaluated a total perfusion deficit (TPD) of ≥ 10% as a more conservative marker of ischemia.

**Results:**

[^15^O]H_2_O PET identified more patients with regional ischemia: 17(46%) vs 9(24%), agreement: 59% corresponding to a Cohen’s kappa of .31 [95%CI .08-.53], (*P* < .001). Using the more conservative TPD ≥ 10%, the agreement increased to 86% corresponding to a kappa of .62 [95%CI .33-.92], (*P* = .001). For the subgroup of patients with no known heart disease (*n* = 18), the agreement was 94%. Interrater agreement was 95% corresponding to a kappa of .89 [95%CI .74-1.00] (*P* < .001).

**Conclusions:**

In clinical transition from ^82^Rb to [^15^O]H_2_O PET, it is important to take into account the higher frequency of patients with regional ischemia detected by [^15^O]H_2_O PET.

**Graphical Abstract:**

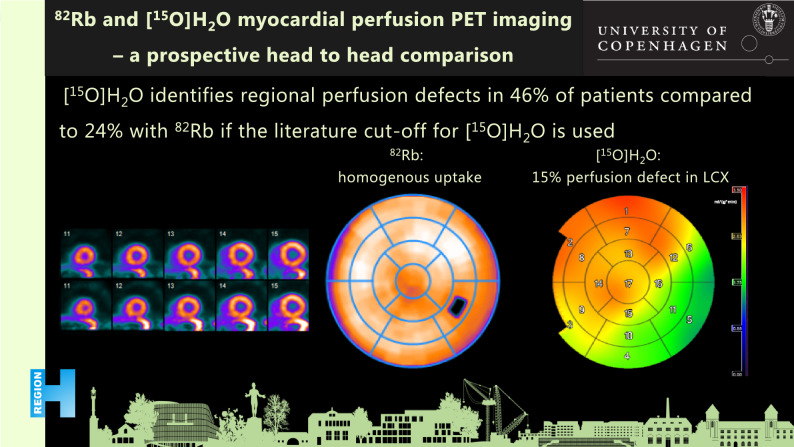

**Supplementary Information:**

The online version contains supplementary material available at 10.1007/s12350-023-03372-7.

## Introduction

Identification of patients with coronary artery disease (CAD) that will most likely benefit from revascularization is guided by non-invasive work-up e.g. myocardial perfusion imaging.^1^ For decades, myocardial perfusion imaging was used with single photon emission computed tomography (SPECT) with e.g. [^99m^Tc]Sestamibi or other irreversible tracers which are extracted into the myocardium dependent on the level of perfusion and not significantly redistributed back into the vascular system. For these irreversible tracers, regionally reduced perfusion during stress but not during rest is referred to as stress-induced ischemia. Reduced perfusion during both stress and rest, matched defects, are considered to be scarring.

A number of sites have switched to PET using ^82^Rb eluted from an ^82^Sr generator. The PET technology results in high-resolution images, and due to the short half-life of ^82^Rb (76 s), rest and stress tests can be performed shortly after each other reducing the entire examination from a two-day protocol to no more than 30 minutes. ^82^Rb is actively transported into the myocytes through the Na^+^/K^+^ pump where the tracer is trapped allowing for static imaging. Hence, the resulting perfusion images are comparable to SPECT images but with better resolution. Further, the software packages for ^82^Rb PET support the quantification of myocardial blood perfusion (MBF). The increase in MBF referred to as the coronary flow reserve (CFR) allows detection of globally reduced perfusion e.g. in triple-vessel disease (balanced ischemia) or microvascular disease.^2^ The quantification method differs between software packages,^3^ but are generally based on a one-tissue compartment model:

1 $$ C_{{\text{T}}} \left( t \right) = K_{1} e^{{ - k_{2} t}} \otimes C_{{\text{A}}} \left( t \right) $$

*C*_T_ is the myocardium time-activity-curve and *C*_A_ is the arterial input function both obtained from the dynamic PET series and ⊗ denotes the convolution operation. *K*_1_ and *k*_2_ are the influx and efflux rate constants. For the irreversible ^82^Rb, *k*_2_ is close to zero. The measured influx *K*_1_ represents MBF and is subsequently corrected for partial volume, motion and arterial blood volume by software package specific corrections.^3^

Another PET perfusion tracer, [^15^O]H_2_O is a cyclotron product with a short physical half-life (122 s) requiring an on-site cyclotron. With the rapidly developing cyclotron technology, our site has established a mini-cyclotron GENtrace (GE healthcare, Uppsala, Sweden) dedicated for production of ^15^O and subsequent synthesis to [^15^O]H_2_O. In contrast to the irreversible PET tracer ^82^Rb, [^15^O]H_2_O is a freely diffusible tracer. The software package calculation for [^15^O]H_2_O is also based on the one-tissue compartment model (Eq. 1) but contrary to ^82^Rb, the efflux rate constant *k*_2_ is used to achieve a more robust estimation for MBF.^4,5^ *k*_2_ represents the clearance of tracer from the tissue and is—at least in theory—more independent of partial volume, motion,^4,6^ and even attenuation correction^5^ which affect only the influx as *k*_2_ is measured relative to the influx. Thus, [^15^O]H_2_O PET allows for parametric images with robust MBF estimates at the expense of static robust high-resolution images obtained using ^82^Rb PET.

While water is freely diffusible with an extraction fraction of 1 in myocardial tissue allowing for direct measurements of perfusion, ^82^Rb has a limited extraction which is reduced at higher perfusion rates and correction for the lower extraction is necessary. The extraction corrections are based on the Renkin-Crone model^7,8^:

2 $$ E = 1 - e^{{ - {\text{PS}}/{\text{MBF}}}} $$

with PS being the permeability-surface product and E the extraction. The ^82^Rb quantification with correction for decreased extraction has been validated against [^15^O]H_2_O PET.^9^ Corrections are typically not applied to the static images used for both visual interpretation and comparison to a normal database but only to the additional quantitative MBF and CFR.

[^15^O]H_2_O has been used mainly in research since the mid 1980’s to quantify MBF. [^15^O]H_2_O PET is quantitatively validated against microspheres in pigs, which is considered the reference standard^10^ and to invasive pressure-measurements in coronary vessels (Fractional Flow Reserve, FFR), thermodilution and has shown prognostic value.^11–13^ In fact, early studies on FFR used [^15^O]H_2_O PET as the reference standard.^14^ For [^15^O]H_2_O PET, the most accurate metric for predicting an FFR-positive coronary stenosis in patients without previous cardiac disease is MBF during stress (MBF_stress_) in two neighboring segments ≤ 2.3 mL/(min·g) in a 17 segment model.^15^

The clinical transition from [^99m^Tc]Sestamibi SPECT to ^82^Rb PET is straightforward with better image quality and patient experience, while the clinical transition to [^15^O]H_2_O PET may be more complicated due to the aforementioned differences in uptake mechanisms. ^82^Rb PET and [^15^O]H_2_O PET are both validated for heart perfusion but have not previously been compared for clinical performance in a prospective cohort of patients suspected for myocardial ischemia. During our clinical transition from ^82^Rb PET to [^15^O]H_2_O PET, we performed a head-to-head comparison in a mixed population with suspected myocardial ischemia and a high fraction of patients with previous heart disease. We hypothesized that there is no significant difference in the overall occurrence of detected clinically relevant myocardial ischemia as determined with ^82^Rb PET and [^15^O]H_2_O PET and that in the vast majority of cases, the patients would be assessed similarly.

## Methods

### Study population

We prospectively and consecutively included patients referred from the Department of Cardiology, Copenhagen University Hospital Bispebjerg from January to April 2022. The study was initiated when [^15^O]H_2_O production was approved at our site and during a four month overlap with continued clinical use of ^82^Rb. Thus all [^15^O]H_2_O PET scans were performed during the startup period. The center has a high-throughput with around 1,500 heart PET examinations per year^16^ and during the startup period, a number of exams were co-reviewed by experts from Turku PET Centre to ensure quality. To increase the likelihood of ischemia in the cohort, we included patients with typical anginal chest pain and with at least one of the following risk factors: a family history of cardiac disease (< 55 years for male and < 65 years for female family members), smoking, diabetes, hypertension, BMI > 30 or hyperlipidemia. Exclusion criteria were unstable angina, significant chronic obstructive lung disease or asthma, claustrophobia, acute severe illness or a significant language barrier. The study was approved by the Research Ethics Committee of the Capital Region of Denmark (ID: H-21016899) and written consent to participate were obtained from all individuals after receiving oral and written information according to the Helsinki declaration. All data were handled according to regulations by The Danish Data Protection Agency.

### [^15^O]H_2_O production

[^15^O]H_2_O was produced in two steps. First, a target gas mixture (97.5% ^15^N_2_, 2.5% O_2_) was continuously bombarded for a few minutes with a 7.8 MeV proton beam in a GenTrace cyclotron (GE, Uppsala, Sweden) dedicated to the production of ^15^O. Second, for administration of [^15^O]H_2_O, the target was mixed with the transport gas (N_2_ with 4% H_2_) and pushed into an oven with a 400 °C hot palladium wire. The resulting radioactive water vapor was fed into a bedside automated production system (Hidex RWG, Hidex Oy, Turku, Finland) consisting of a dual membrane system to mix physiological saline with [^15^O]H_2_O. The resulting radioactive saline solution was injected into the patient without further user interaction.

### PET scans

All subjects refrained from using caffeine-containing beverages and food or theophylline-containing medication for 24 hours before examination. Furthermore, phosphodiesterase type 5 inhibitors were withheld five days before examination, antithrombotic medicine containing Dipyridamole or Nicorandil two days before, extended-release nitrates 12 hours before and short-acting nitroglycerin two hours before examination. All patients were scanned using a Discovery 710 PET/CT scanner (GE Healthcare, Milwaukee, WI, USA). After a CT scan for attenuation correction and for anatomical localization, a 5 minutes dynamic emission scan in list mode was performed during resting condition after intravenous injection of 1100 MBq of ^82^Rb eluted from an ^82^Sr/^82^Rb generator (CardioGen-82; Bracco, Princeton, NJ). [^15^O]H_2_O PET in resting condition was mean performed earliest 10 minutes after ^82^Rb PET, and a dose of 394 MBq (range: 345-563 MBq) [^15^O]H_2_O was injected intravenously using a synthesis and injection system, Hidex RadioWaterGenerator (Hidex, Turku, Finland) and a 5-minutes scan was initiated simultaneously with the bolus arrival. After a 10 minutes interval to allow for decay of radioactivity, an identical PET sequence was performed during stress conditions induced by intravenous adenosine infusion (140 μg/kg/min) for 6 minutes. Adenosine was started 2 minutes prior to the stress PET scans to achieve maximum hyperaemia. We did not randomize the order of ^82^Rb and [^15^O]H_2_O PET as several patients could not cooperate to two adenosine infusions and we aimed to ensure a clinically useful ^82^Rb PET examination. Reconstruction of dynamic PET images was performed using ordered subset expectation maximization (OSEM) with Time of Flight (ToF) (2 iterations, 24 subsets and 6.4 mm in-plane filtering). For ^82^Rb a static reconstruction for the last 150 s was used. The Corridor 4DM software version 2018 (Invia Medical Imaging Solutions, Ann Arbor, MI, USA) was used for the analysis of ^82^Rb PET data, while CarimasCE software version 1.3.1. (Turku, Finland) was used for [^15^O]H_2_O PET data. Corridor 4DM estimates quantitative MBF from ^82^Rb *K*_1_ measurements using a modified version of Equation 2 according to Lortie^17^:

3 $$ K_{1} = MBF \cdot \left( {1 - .77 \cdot e^{{ - \frac{.63}{{MBF}}}} } \right) $$

### PET interpretation

#### ^82^Rb PET

^82^Rb PET was evaluated as part of daily clinical routine by a nuclear medicine specialist with > 10 years of experience in myocardial imaging. Previous studies have shown good inter-observer agreement.^18,19^ ^82^Rb PET were assessed visually using the ‘splash’ images using polar plots with relative differences compared to a normal database. The degree of relative defects within the myocardium was rated using 17 segments and 4 degrees of reduction, i.e. maximum total score 68. A stress defect score of 7 or more (i.e. ≈10%) of the myocardial wall was considered significant. Based on the clinical readings, the ^82^Rb PET was classified into four groups (normal, regional ischemia, globally reduced myocardial perfusion and myocardial scarring, see Table 1 for definitions). To further simplify data, the classifications were reduced to two groups of normal and regional ischemia, the latter included global reduction with suspicion of triple-vessel disease. The vascular territory involved was noted as left anterior descending artery (LAD), left circumflex artery (LCX) or right coronary artery (RCA).

**Table 1:** Definitions of classifications

	4 group classification				Maximal score	2 group classification	
	Normal	Regional ischemia	Global reduction	Scarring		Normal	Regional ischemia
^82^Rb	Stress score defect <7	Focal stress score defect ≥ 7	CFR<1.8	Matched defects	17 segments and 4 degrees of reduction, i.e. maximum total score 68.	Stress score defect <7	Focal stress score defect ≥ 7 or suspicion of triple-vessel disease
[^15^O]H_2_O	MBF_stress_ > 2.3 mL/(min·g)	≥ 2 neighboring segments with MBF_stress_≤ 2.3 mL/(min·g)	MBF_stress_ ≤ 2.3 mL/(min·g)	Matched defects	17 segments and 4 degrees of reduction, i.e. maximum total score 68.	Stress score defect <7	Focal stress score defect ≥ 7 or suspicion of triple-vessel disease

[^15^O]H_2_O stress scores: MBF_stress_ > 2.3: score 0; 2.0 > MBF_stress_ ≤ 2.3: score 1; 1.7 < MBF_stress_ ≤ 2.0: score 2; 1.4 < MBF_stress_ ≤ 1.7: score 3; MBF_stress_ ≤ 1.4: score 4

#### [^15^O]H_2_O PET

[^15^O]H_2_O PET was assessed according to Danad^15^ with a cut-off of two neighboring segments with MBF_stress_≤ 2.3 mL/(min·g). Additionally, in line with ^82^Rb, total perfusion deficit (TPD) was calculated as a score of segmental reduction in percentage according to the following limits: normal (MBF_stress_ > 2.3; score 0), mildly reduced (2.0< MBF_stress_ ≤ 2.3; score 1), moderately reduced (1.7< MBF_stress_ ≤ 2.0; score 2), severely reduced (1.4< MBF_stress_ ≤ 1.7; score 3), and very severely reduced (MBF_stress_ ≤ 1.4; score 4). TPD ≥ 10% was tentatively considered significant for ischemia. TPD is not a validated measure in [^15^O]H_2_O PET but was introduced in our clinic during the training period as we quickly realized that the threshold of two segments ≤ 2.3 mL/(min·g) identified too many patients with ischemia in the current patient population, which was also demonstrated in a recent study in patients with previous myocardial infarction or PCI.^20^ All [^15^O]H_2_O PET images were evaluated after the startup period had ended and [^15^O]H_2_O PET had been clinical routine for 2 months. Readers had > 10 years of experience in nuclear cardiology (UT and MK). [^15^O]H_2_O PET images were randomized and readers were blinded to the result of the ^82^Rb PET but with full access to all clinical data to simulate daily clinical routine. As for ^82^Rb, each [^15^O]H_2_O PET examination was classified into four groups based on the two segment threshold with MBF_stress_≤ 2.3 mL/(min·g) and two groups based on the aforementioned TPD of 10% (see Table 1 for definitions). The involved vascular territories (LAD, LCX and/or RCA) were noted. In case of discrepancy between the readers, consensus reading was performed. If a scan was classified with both a regional ischemia and global perfusion reduction or scarring, the regional defect overruled the other findings. To assess how previous heart disease affected the agreement, we performed a subgroup analysis of patients without known heart disease.

### Statistics

Data are reported with mean and standard deviation or median and interquartile range. Differences in heart rate response during adenosine infusion were compared using a paired Student’s t-test. Using SPSS (IBM SPSS Statistic version 25). Agreement was defined as the number of identical classification divided by the total number of patients. Cohen’s kappa was used to compare agreement between methods and between readers for the two and four group comparisons when sample sizes were sufficient. Kappa-values were evaluated according to Altman: *κ* ≤ .2: poor, .2 < *κ* < .4: fair, .4 < *κ* < .6: moderate, .6 < *κ* < .8: good, *κ* > .8: very good.^21^ McNemar’s test were used to test for agreement between tracers with the 2 × 2 contingency tables. Pearson’s correlation coefficient was used to compare the measured perfusion with the two methods. The *K*_1_ values were calculated from ^82^Rb MBF estimates using Equation 3 to compare the apparent ^82^Rb uptake in static images to the parametric [^15^O]H_2_O PET MBF images.

## Results

A total of 57 patients were included. A number of subjects were subsequently excluded due to cancellation (*n* = 1), [^15^O]H_2_O production failure (*n* = 3), intravenous access failure (*n* = 1), not able to receive adenosine twice (*n* = 4), or technical failure (*n* = 5) leaving 43 patients for the analysis. During reading, 6 additional examinations were excluded due to excessive movement artefacts (*n* = 4) or insufficient vasodilatation (*n* = 2) leaving 37 patients with diagnostic scans with both tracers (Suppl. Figure 1). Table 2 lists demographic and clinical data of the included patients. Please note the high number of patients with significant previous heart disease. Infusion of adenosine elicited a heart rate response from on average 67.0 (± SD 9.1) to 89.6 (± 12.1) during ^82^Rb scan and a slightly diminished (*p *< .005) response from 69.6 (± 11.7) to 87.8 (± 13.1) during the [^15^O]H_2_O scan, which was probably due to habituation.

**Table 2:** Demographic and clinical data of the 37 included patients

Age (years)	66 (IQR: 59–74)
Sex	30 males (80%)
Body weight (kg)/BMI (kg/m^2^)	86 (SD:18)/28 (SD:4.8)
Atrial fibrillation during exam	2 (5%)
Previous CAD—PCI/CABG/AMI	18 (49%)—14/3/8
Previous heart disease	20 (54%)
Calcium score (no previous PCI)	180 (19 available, IQR: 50–930)
EDV (mL/m^2^)	52 (SD: 14.7)
LVEF (%)	56 (SD: 11)
LVEF < 45%	6 (16%)

*IQR*, interquartile range; *BMI*, body mass index; *CAD*, cardiovascular disease; *PCI,* percutaneous coronary intervention; *CABG,* coronary artery bypass graft surgery; *EDV*, end-diastolic volume normalized to body surface; *LVEF,* left ventricle ejection fraction

Previous heart disease includes: heart failure (LVEF<50%), atrial fibrillation, second or third degree atrioventricular block, (acute) myocardial infarction, PCI or CABG

Please refer to Table 3 for comparison of the ^82^Rb PET and consensus [^15^O]H_2_O PET ratings for the entire group and Table 4 for patients without previous heart disease. We found more positive findings with [^15^O]H_2_O than ^82^Rb PET as ^82^Rb PET identified 9 (24%) patients with regional ischemia vs 17 (46%) patients by [^15^O]H_2_O PET. Of the 9 positive findings with ^82^Rb PET only one was negative with [^15^O]H_2_O PET. Agreement between the tracers when rating into four groups was 59% corresponding to a fair kappa of .31 [95%CI .08-.53], (*P* < .001) for the entire population (Table 3A), and 72% for patients with no previous heart disease (Table 4A).

**Table 3:** Agreement between ratings based on [^15^O]H_2_O and ^82^Rb for the entire population

A	[^15^O]H_2_O				Total
^82^Rb	Normal	Regional ischemia	Global reduction	Scarring	
Normal	**14**	7	2		23
Regional ischemia	1	**8**			9
Global reduction		2			2
Scarring	3				3
**Total**	18	17	2		37

B	[^15^O]H_2_O	
^82^Rb	No regional defect	TPD > 10%
No regional defect	**26**	4
Regional defect > 10%	1	**6**

Bold represents identical ratings. A: Agreement between the tracers when rating into four groups. Note that three patients were classified with scarring using ^82^Rb PET and none using [^15^O]H_2_O PET. B: Rating into two groups with and without a TPD > 10%. For discrepancies, see Table 5

*TPD*, total perfusion defect (see methods for definition).

**Table 4:** Agreement between ratings based on [^15^O]H_2_O and ^82^Rb for patients with no prior heart disease

A	[^15^O]H_2_O				Total
^82^Rb	Normal	Regional ischemia	Global reduction	Scarring	
Normal	**11**	3			14
Regional ischemia		**2**			2
Global reduction		1			1
Scarring	1				1
**Total**	12	6			18

B	[^15^O]H_2_O	
^82^Rb	No regional defect	TPD > 10%
No regional defect	**15**	1
Regional defect > 10%	1	**1**

Bold represents identical ratings. A: Agreement between the tracers when rating into four groups. B: Rating into two groups with and without a TPD > 10%.

*TPD*, total perfusion defect (see methods for definition)

Rating into two groups with and without a TPD > 10%, the number of positive findings was reduced from 17 to 10 for [^15^O]H_2_O PET and from 9 to 8 for ^82^Rb PET, indicating the importance of selecting a proper cut-off. The agreement increased to 86% for the entire population corresponding to a good kappa of .62 [95%CI .33-.92], (*P* = .001) (Table 3B) and 94% for the patients with no previous heart disease (Table 4B). No significant differences between tracers was found for the entire population or the patients with no previous heart disease (McNemar, *P* = .16 and *P* = .50, respectively). A total of six patients had a TPD > 10% for both tracers and five of these had identical scoring of vascular territory.

**Figure 1:**
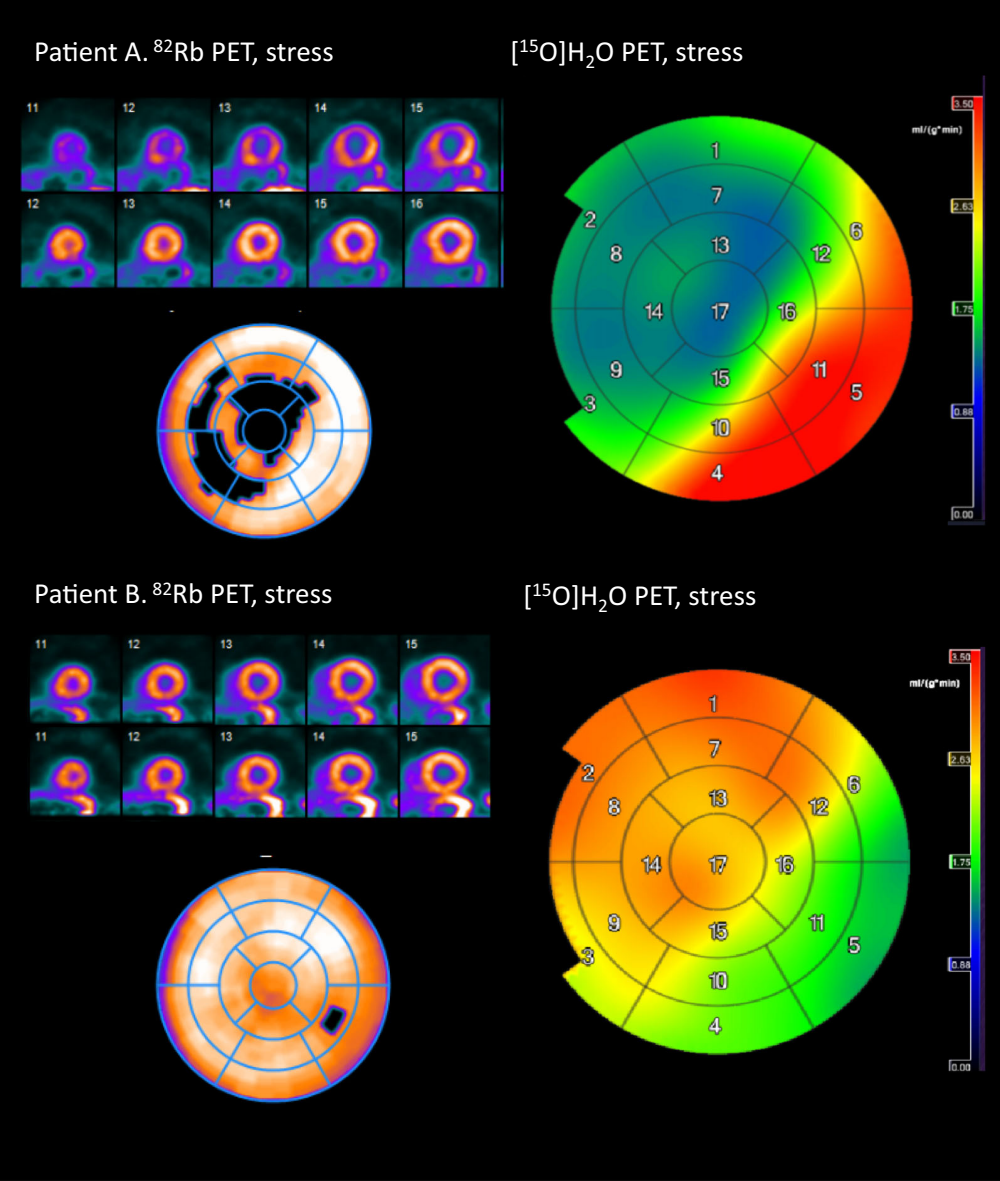
Patient A: Example of agreement. 61 year old man with angina but without prior known CAD. Both tracers showed large perfusion defects (^82^Rb: 25% and [^15^O]H_2_O: 54%) during stress in LAD and RCA. Patient B. Example of disagreement. 74 year old man with angina, a number of risk factors for CAD and dilated left atrium. ^82^Rb showed homogenous tracer uptake while [^15^O]H_2_O showed a defect of 15% in LCX and/or RCA

Table 5 shows the cases with discrepancies, and Figure 1 shows examples of agreement and disagreement. The inter-reader agreement of [^15^O]H_2_O PET was 84% for four group classification corresponding to a good kappa of .73 [95%CI .54-.91] (*P* < .001), and for classification in two groups, the agreement was 95% corresponding to a very good kappa of .89 [95%CI .74-1.00] (*P* < .001).

**Table 5:** Discrepancies from Table 3

^82^Rb	[^15^O]H_2_O	# of patients	Explanations/clinical findings	Follow-up (12 months)
Normal	Regional defect	7	4 had minor defects of 3-9% not visible with ^82^Rb.	2 had repeated contacts due to chest pain with 1 having ICA without intervention.
			1 had TPD > 10% (15%): Male, 74 years. No prior CAD. Multiple risk factors and arterial fibrillation. LVEF: 45%, EDV: 79 mL/m^2^. Calcium score 1449.	Followed for atrial fibrillation. No contacts due to chest pain. Figure 1, patient B.
			1 had TPD > 10% and global defect: Male, 58 years. Triple bypass. B-cell lymphoma, hypothyroidism. LVEF: 53%, EDV: 72 mL/m^2^.	Persistent chest pain.
			1 had TPD > 10% (16%): Male, 51 years. PCI twice. Increasing frequency of chest pain. LVEF: 61%, EDV: 58 mL/m^2^.	No contacts due to chest pain.
Normal	Global reduction	2	Global reductions explained by atrial fibrillation or reduced LVEF.	Heart amyloidosis, cardiogenic shock, LVEF: 25%.
				Treated for arrhythmia.
Regional	Normal	1	^82^Rb Regional defect of 5-10%.	No subsequent ICA.
Global reduction	Regional defect	2	1 had minor regional defect of 7% + global reduction with [^15^O]H_2_O.	Repeated chest pain. ICA one year later showed diffuse atheromatosis.
			1 had TPD > 10% (21%): Female, 65 years. Bypass, bicuspid aorta valve, rheumatoid arthritis, increasing dyspnoea, LVEF: 69%, EDV: 50 mL/m^2^.	Less dyspnea, blood percent increased. No contacts due to chest pain.
Scarring	Normal	3	1 had previous PCI (RCA) due to AMI, ^82^Rb defect in LCX.	No contacts due to chest pain.
			1 had previous PCI (RCA) due to AMI with inferior hypokinesia, ^82^Rb defect in RCA.	
			1 had no previous CAD, slight diastolic dysfunction. Interpreted as atheromatosis	
Table 3B	Table 3B	1	^82^Rb defect: 25%, [^15^O]H_2_O defect: 7%.	ICA: diffuse atheromatosis. Aortic valve implantation complicated by cerebral embolism.
Regional defect > 10%	TPD<10%		Female, 81 years. Breast cancer, hypertension, chest pain, LVEF: 60%, EDV: 72 mL/m^2^, Calcium score 499.	

*ICA*, invasive coronary angiography; *TPD*, total perfusion deficit; *CAD*, coronary artery disease; *LVEF*, left ventricle ejection fraction; *EDV*, end diastolic volume; *PCI*, percutaneous coronary intervention; *LCX*, left circumflex artery; *RCA*, right coronary artery; *AMI*, acute myocardial infarction

**Figure 2:**
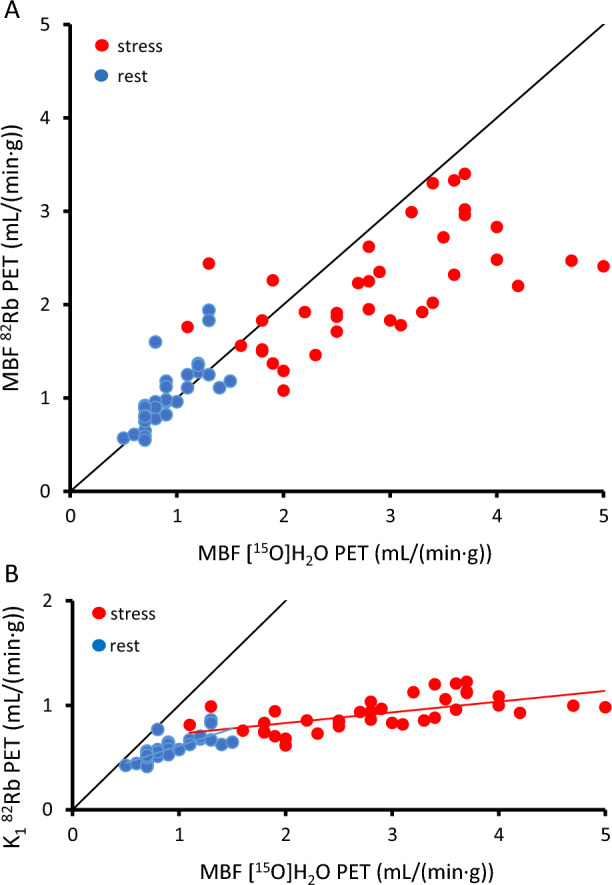
**A** Correlation between myocardial blood flow (MBF) measured with ^82^Rb as a function of MBF measured with [^15^O]H_2_O. (Rest: *r* = .75 [95%CI .56-.86], *P* < .000001; stress: *r* = .62 [95%CI .37-.79], *P* < .0001). The ^82^Rb measures are corrected for a flow-dependent lower extraction according to Lortie ^17^. B: Correlation between uncorrected ^82^Rb *K*_1_ and [^15^O]H_2_O MBF values (rest: *r* = .77 [95%CI .59-.87], slope = .33, *P* < .0000001; stress: *r* = .62 [95%CI .38-.79], slope = .10, *P* < .0001). Note the clear bias with lower perfusion values using ^82^Rb, even when correcting for reduced extraction in A. Blue represent resting conditions and red represent stress conditions

MBF obtained with the two methods correlated significantly but lower values were measured at high perfusion for ^82^Rb compared to [^15^O]H_2_O—even after correction for the lower extraction of ^82^Rb (see Figure 2): Figure 2a: Rest: *r* = .75 [95%CI .56-.86], *P* < .000001; stress: *r* = .62 [95%CI .37-.79], *P* < .0001; and Figure 2b for uncorrected ^82^Rb *K*_1_ and [^15^O]H_2_O MBF values: Rest: *r* = .77 [95%CI .59-.87], slope = .33, *P* < .0000001 and stress: *r* = .62 [95%CI .38-.79], slope = .10, *P* < .0001.

**Figure 3:**
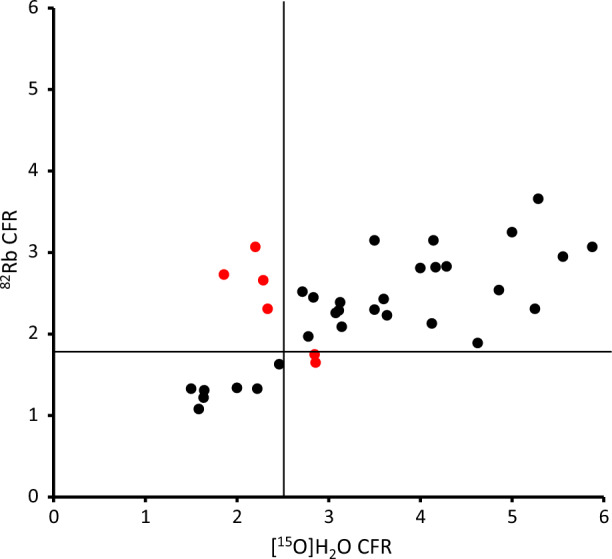
CFR compared for the two tracers (*r* = .66 [95%CI .42-.81], *P* < .0001). Note that the cut-offs for reduced CFR are different^15^. It is noteworthy that four subjects (marked red) were regarded normal according to the ^82^Rb scan and had too low CFR according to the [^15^O]H_2_O scan, and additional two subjects (also marked red) were opposite below normal for ^82^Rb but not for [^15^O]H_2_O

The CFR obtained with the two tracers also correlated significantly (Figure 3) (*r* = .66 [95%CI .42-.81], *P* < .0001) and also with systematically lower values for ^82^Rb compared to [^15^O]H_2_O. Note that the cut-off from the literature as depicted by the solid lines is also lower for ^82^Rb. A total of 6 patients (marked red) would be classified differently in terms of CFR by the two tracers and one of these had different final ratings.

## Discussion

This is the first direct comparison of myocardial perfusion PET with ^82^Rb and [^15^O]H_2_O for diagnosing obstructive CAD in a mixed population of patients with more than 50% having previous heart disease. Only fair agreement (*P* < .001) was found with [^15^O]H_2_O PET reporting ischemia more frequently than ^82^Rb PET. Applying a TPD cut-off of 10% improved agreement with ^82^Rb but still identified three more subjects with ischemia.

Part of the disagreement between ^82^Rb and [^15^O]H_2_O can be explained by the application of the cut-off of 2.3 mL/(min·g) which was determined in patients without prior CAD^15^ that were slightly younger (61 vs 66 years), weighed less (80 vs 86kg) and included more women (42% vs 20%). Thus, the cut-off is likely to identify too many patients with ischemia in a mixed population. This finding is in accordance with the PACIFIC 2 study,^20^ showing reduced specificity of [^15^O]H_2_O PET in patients with previous ischemic heart disease. However, in the subpopulation of the patients with no previous heart disease, [^15^O]H_2_O PET also seem to find regional ischemia more frequently than ^82^Rb PET (Table 4A). The solely use of relative perfusion defects for ^82^Rb may be less sensitive. We do not know to what degree [^15^O]H_2_O PET identifies patients with signs of hemodynamically significant coronary artery stenosis as determined by invasive coronary angiography (ICA) since only patients with stress-induced defects on ^82^Rb PET were considered for ICA. None of the patients identified with perfusion defects by [^15^O]H_2_O PET who had a normal ^82^Rb PET scan had subsequent angiographic imaging or were subsequently hospitalized suspected for myocardial infarction but the limited number of patients and short time of follow-up of 12 months does not rule out that the patients did indeed have significant coronary artery stenosis (see Table 5 for details of the patients with discrepancy). We anticipate that a less dichotomous interpretation of [^15^O]H_2_O PET than simply applying an MBF cut-off value of 2.3 mL/(min·g) will be needed in a mixed population, e.g. taking into account other factors potentially contributing to globally decreased stress myocardial perfusion such as previous revascularization, atrial fibrillation, decreased left ventricular ejection fraction, diabetes, and other conditions involving microvascular dysfunction. Segmental relative CFR calculation could be a possible tool to evaluate perfusion in these patients.^22^

^82^Rb has a flow-dependent lower extraction of tracer compared to the freely diffusible [^15^O]H_2_O. This is partly corrected in the quantitative measures, achieving a high correlation between MBF and CFR measured with the two methods (Figures 2, 3), although a flow-dependent bias is still clearly visible in Figure 2a. The bias in our study is more pronounced than previous studies have reported^23^ and must be kept in mind when comparing MBF measured with the two methods. But more importantly, the ^82^Rb images which are visually assessed using ‘splash’ images or polar plots are indeed images of tracer uptake, i.e. uncorrected *K*_1_ values which show a much more pronounced bias. Figure 2b shows that differences in ^82^Rb *K*_1_ correspond to much larger differences in the *k*_2_-derived MBF using [^15^O]H_2_O, especially for stress images, which have a slope of only .10, i.e. visual differences are about 10 times greater for [^15^O]H_2_O compared to ^82^Rb. This is not only true between subjects as in Figure 2b but also within subjects. Thus, the higher extraction likely explains the higher frequency of patients with regional ischemia detected by [^15^O]H_2_O.

It was shown by Danad et al^15^ that rest images of [^15^O]H_2_O PET were not prognostic in a setting of patients with no prior heart disease. Indeed, in some sites only stress images are obtained resulting in a highly efficient workflow. At our own institution, half of our patients had previous heart disease (Table 2) and traditionally, rest ^82^Rb images were used to differentiate ischemia from previous infarcts/fibrosis/scarring. Two factors reduce the clinical usefulness of rest [^15^O]H_2_O images for differentiating between scarring and ischemia. Firstly, as explained above, [^15^O]H_2_O PET shows higher variation in MBF and differentiating between true infarcts and minor relative changes may be difficult. Secondly, and more importantly, the efflux constant *k*_2_ is used instead of the influx constant *K*_1_ to assess MBF (see Equation 1). While the influx *K*_1_ is highly sensitive not only to movement and attenuation artefacts but also to tissue defects and old infarcts, *k*_2_ is a rate constant for tracer leaving myocytes, i.e. independent of infarcts and non-perfused areas. [^15^O]H_2_O PET robustly measures the perfusion of the remaining viable tissue at the expense of obscuring areas with partial fibrosis/subendocardial infarcts. Indeed, in our material three subjects were classified with scarring using ^82^Rb, while appearing normal on resting [^15^O]H_2_O PET (Table 3a). It is, however, not known to which degree true infarctions were present. A tissue fraction measure (perfusable tissue fraction, PTF) may be able to identify areas of non-perfused tissue^6,24,25^ but this has not been thoroughly validated in a population with different risk and probability for CAD and did not seem robust in our sample. Thus, PTF was not included in the present study. We found high to very high agreement between readers of [^15^O]H_2_O PET, suggesting a robust assessment tool, although the agreement must be interpreted with the notion that a number of subjects were excluded due to noisy data or motion artefacts. This may be attributed to the patients receiving two rather unpleasant infusions of adenosine combined with less routine by the technical staff in the startup period as we currently rarely experience such artefacts. Limitations include the lack of a reference standard. A reference standard as ICA with FFR measurements would result in a more selected population while a reference standard involving clinical follow-up would require a much larger sample than was possible in the present setting. Sample size is limited as a large number of patients were excluded due to production failure or noisy [^15^O]H_2_O data which was not possible to interpret and we cannot rule out that agreement would been different if the excluded cases were part of the analysis. Based on the experience, we have now applied and received permission to increase the standard injected [^15^O]H_2_O dose to 600 MBq. The population was mixed with a high fraction of patients having had previous CAD with PCI or coronary by-pass surgery, which diminished agreement between the two methods. Indeed, patients were only included if they had relatively high pre-test probability of ischemia to ensure a data set with a considerable number of patients with ischemia. Lack of randomization of the order of ^82^Rb and [^15^O]H_2_O PET is another limitation as the stress period of the first adenosine infusion may interfere with physiological reactions to the second infusion, which may limit the quantitative comparisons of perfusion between the methods.

## New knowledge gained

In a clinical setting, we found only moderate agreement between [^15^O]H_2_O and ^82^Rb perfusion PET as [^15^O]H_2_O PET identifies a higher frequency of patients with regional ischemia.

## Conclusion

[^15^O]H_2_O perfusion PET is a sensitive imaging modality for myocardial ischemia with a high interrater agreement. However, a number of differences exist between ^82^Rb and [^15^O]H_2_O perfusion PET. Primarily, we found only a moderate agreement as [^15^O]H_2_O PET identifies a higher frequency of patients with regional ischemia especially if using the literature cut-off of 2.3 mL/(min·g) determined in individuals without prior CAD. An improved agreement was found using the more conservative TPD of ≥ 10% but future studies are warranted to establish [^15^O]H_2_O PET interpretation criteria in a mixed population. Secondly, matched rest and stress perfusion defects using ^82^Rb interpreted as scarring are not always detected by [^15^O]H_2_O PET which likely detects only transmural scars.

### Supplementary Information

Below is the link to the electronic supplementary material.

Supplementary file1 (DOCX 66 kb)

Supplementary file2 (PPTX 470 kb)

## Data Availability

Anonymised datasets generated during and/or analysed during the current study are available from the corresponding author on reasonable request.

## Abbreviations

CAD: Coronary artery disease
PCI: Percutaneous coronary intervention
CABG: Coronary artery bypass grafting
ICA: Invasive coronary angiography
SPECT: Single photon emission computed tomography
PET: Positron emission tomography
CFR: Coronary flow reserve
MBF: Myocardial perfusion
MBF_stress_: Myocardial perfusion during adenosine vasodilation
TPD: Total perfusion deficit
