# Supplementary figures and images for: 82Rb and [15O]H2O myocardial perfusion PET imaging: a prospective head to head comparison
Source: J Nucl Cardiol. 2023 Oct 3;30(6):2790–802. doi: 10.1007/s12350-023-03372-7 (PMC10682292; doi:10.1007/s12350-023-03372-7)

# **Inclusion diagram**


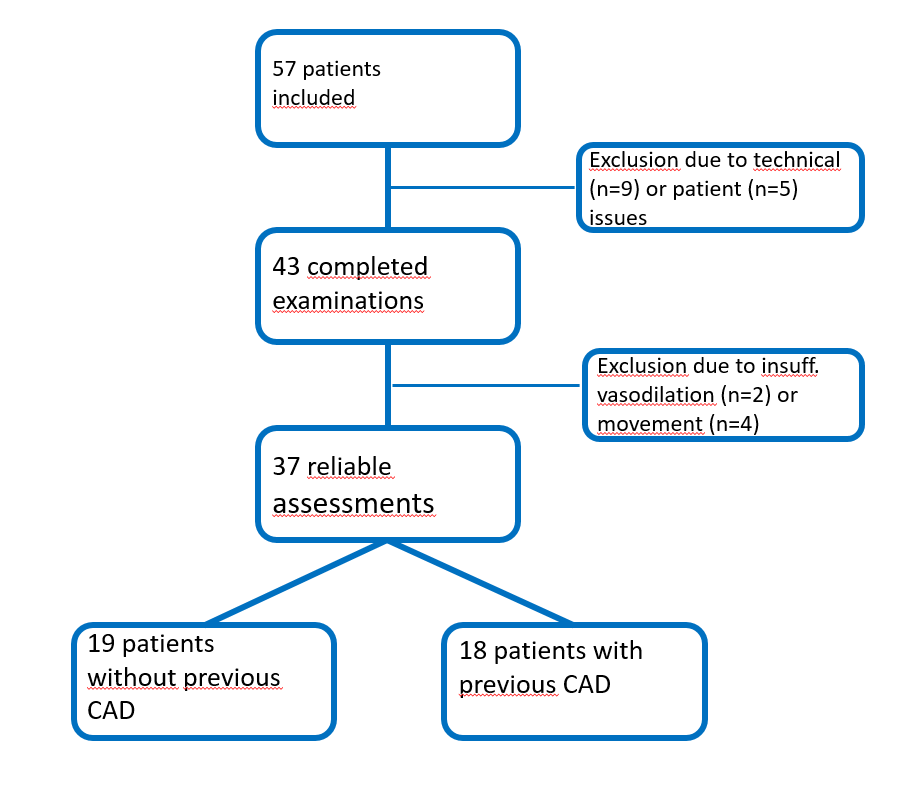

Supplement: Supplementary file 1 — Supplementary file1 (DOCX 66 kb) [file 12350_2023_3372_MOESM1_ESM.docx]
