# Supplementary material for: 82Rb and [15O]H2O myocardial perfusion PET imaging: a prospective head to head comparison
Source: J Nucl Cardiol. 2023 Oct 3;30(6):2790–802. doi: 10.1007/s12350-023-03372-7 (PMC10682292; doi:10.1007/s12350-023-03372-7)
Supplement: Supplementary file 2 — Supplementary file2 (PPTX 470 kb) [file 12350_2023_3372_MOESM2_ESM.pptx]

## Slide 1
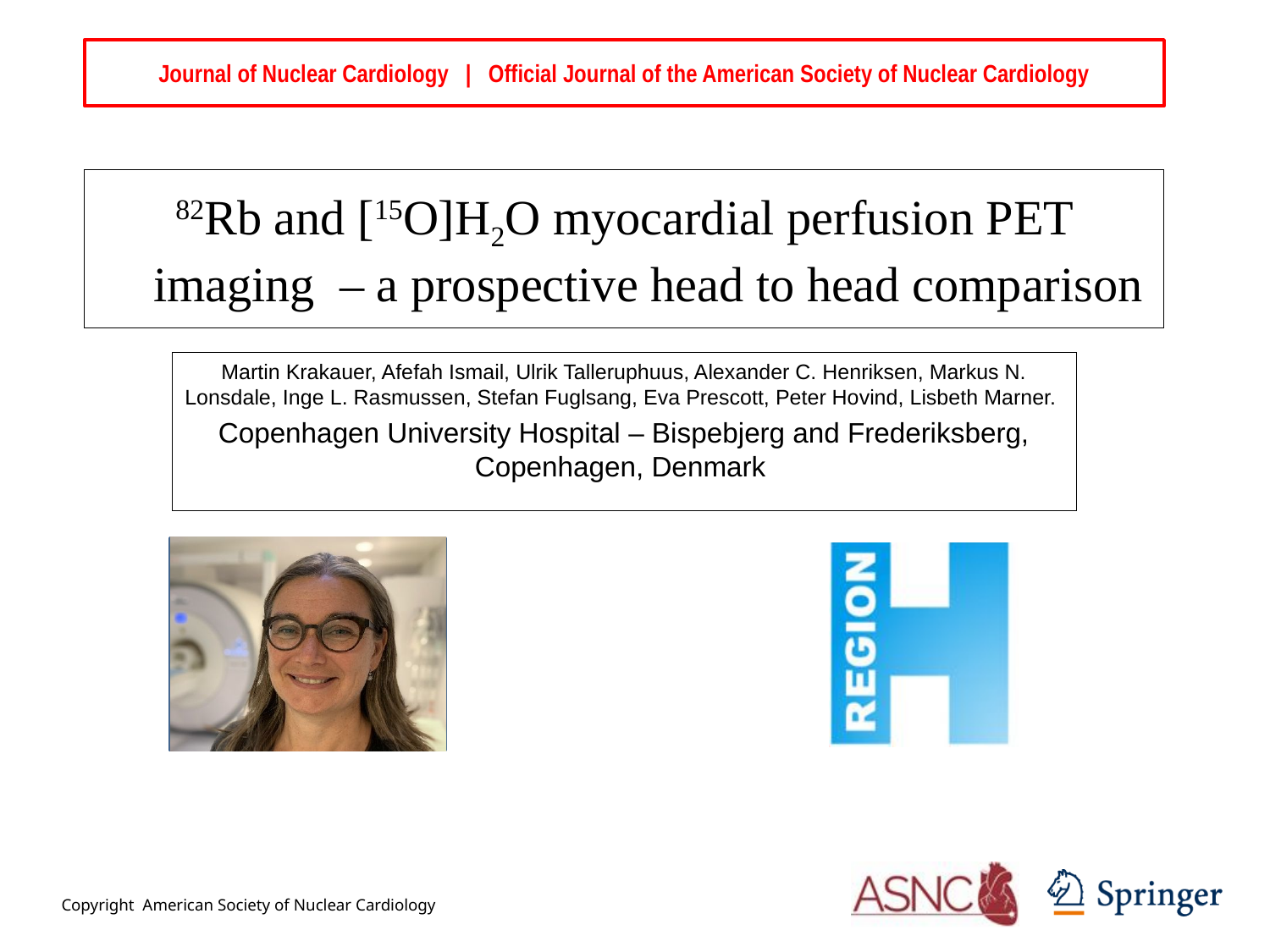

Journal of Nuclear Cardiology | Official Journal of the American Society of Nuclear Cardiology
# 82Rb and [15O]H2O myocardial perfusion PET imaging – a prospective head to head comparison
Martin Krakauer, Afefah Ismail, Ulrik Talleruphuus, Alexander C. Henriksen, Markus N. Lonsdale, Inge L. Rasmussen, Stefan Fuglsang, Eva Prescott, Peter Hovind, Lisbeth Marner.
Copenhagen University Hospital – Bispebjerg and Frederiksberg, Copenhagen, Denmark
Head shot of author
required
Copyright American Society of Nuclear Cardiology

## Slide 2
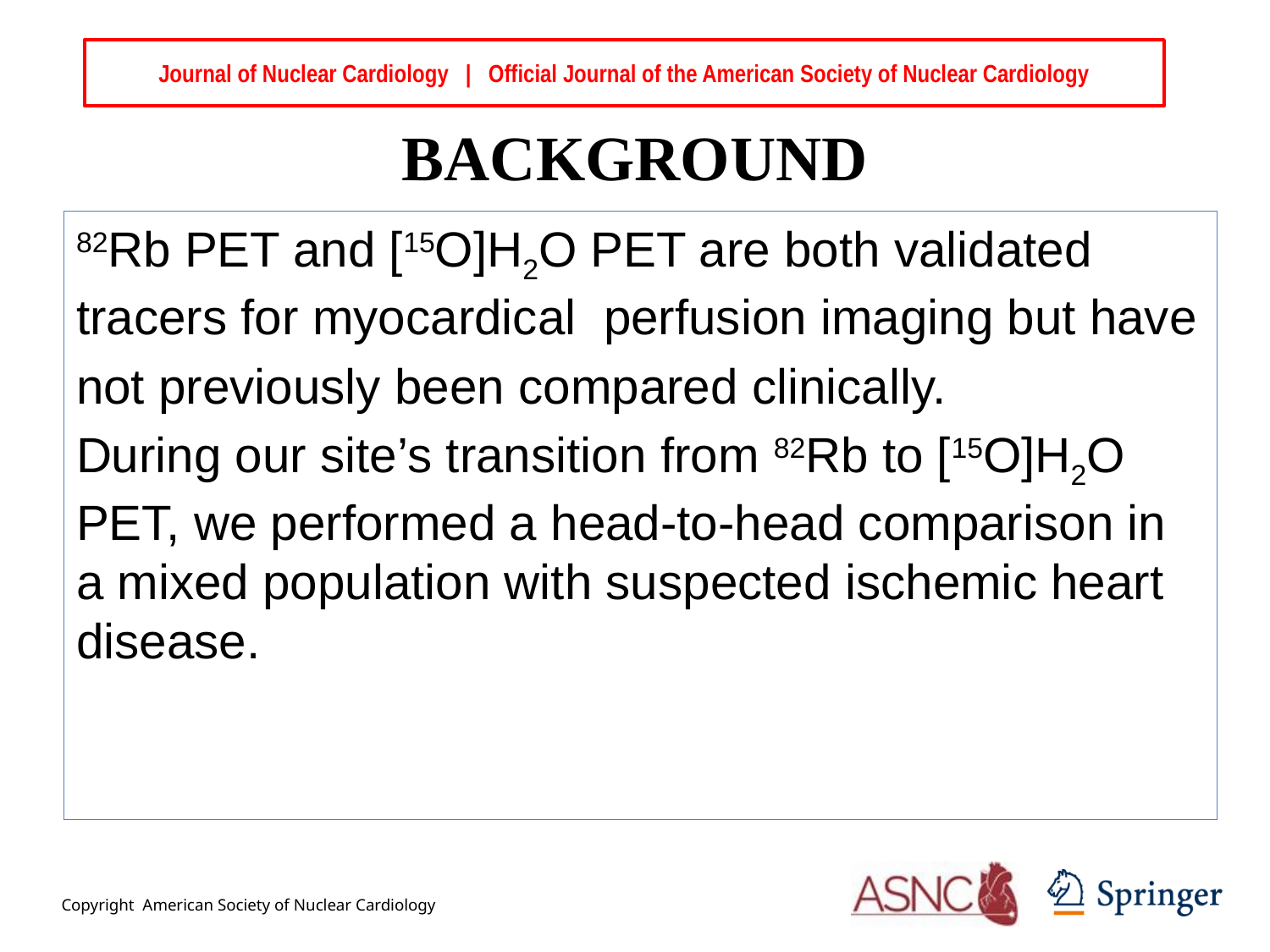

Journal of Nuclear Cardiology | Official Journal of the American Society of Nuclear Cardiology
# BACKGROUND
82Rb PET and [15O]H2O PET are both validated tracers for myocardical perfusion imaging but have
not previously been compared clinically.
During our site’s transition from 82Rb to [15O]H2O PET, we performed a head-to-head comparison in a mixed population with suspected ischemic heart disease.
Copyright American Society of Nuclear Cardiology

## Slide 3
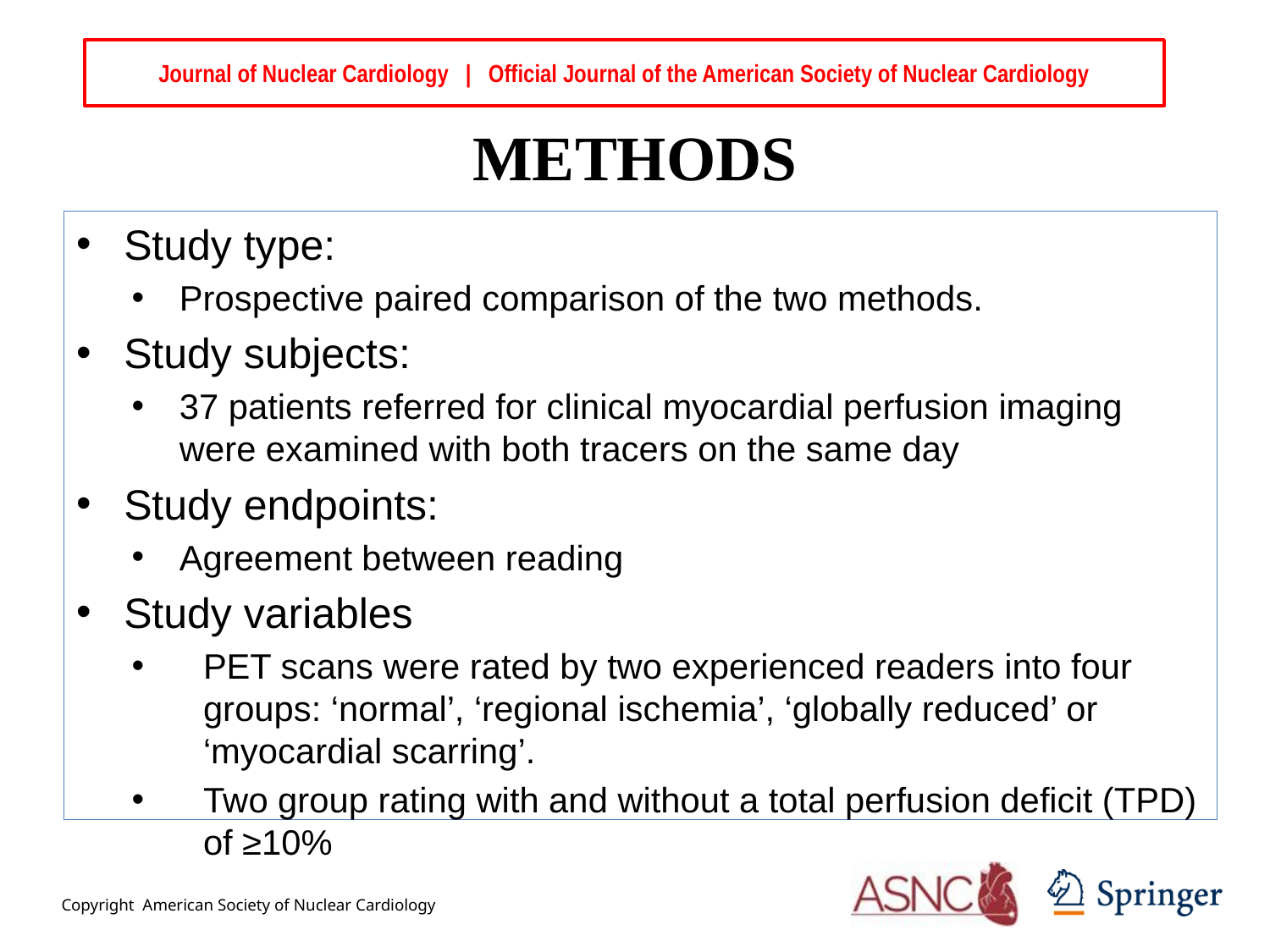

Journal of Nuclear Cardiology | Official Journal of the American Society of Nuclear Cardiology
# METHODS
Study type:
Prospective paired comparison of the two methods.
Study subjects:
37 patients referred for clinical myocardial perfusion imaging were examined with both tracers on the same day
Study endpoints:
Agreement between reading
Study variables
PET scans were rated by two experienced readers into four groups: ‘normal’, ‘regional ischemia’, ‘globally reduced’ or ‘myocardial scarring’.
Two group rating with and without a total perfusion deficit (TPD) of ≥10%
Copyright American Society of Nuclear Cardiology

## Slide 4
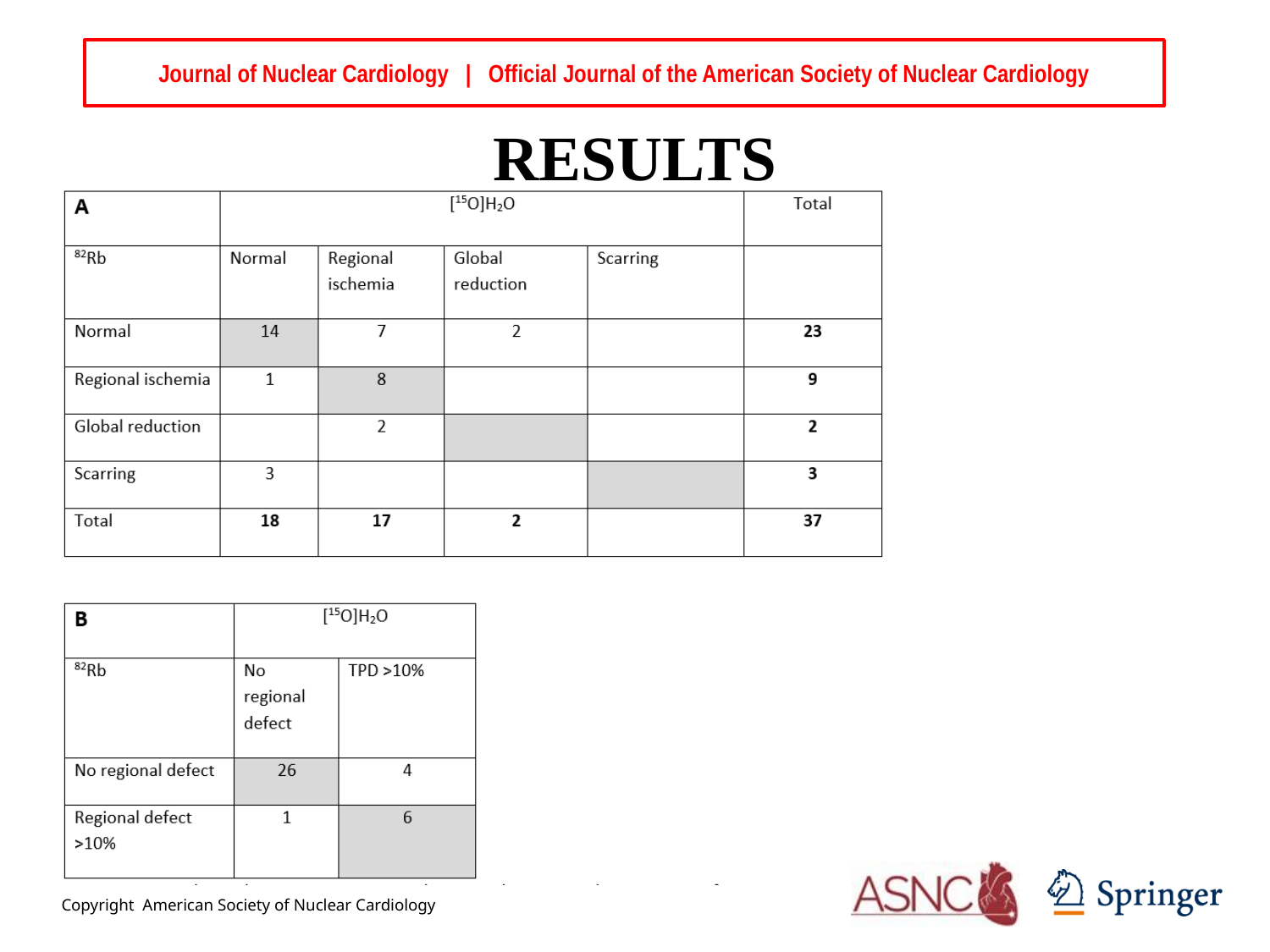

Journal of Nuclear Cardiology | Official Journal of the American Society of Nuclear Cardiology
# RESULTS
Copyright American Society of Nuclear Cardiology

## Slide 5
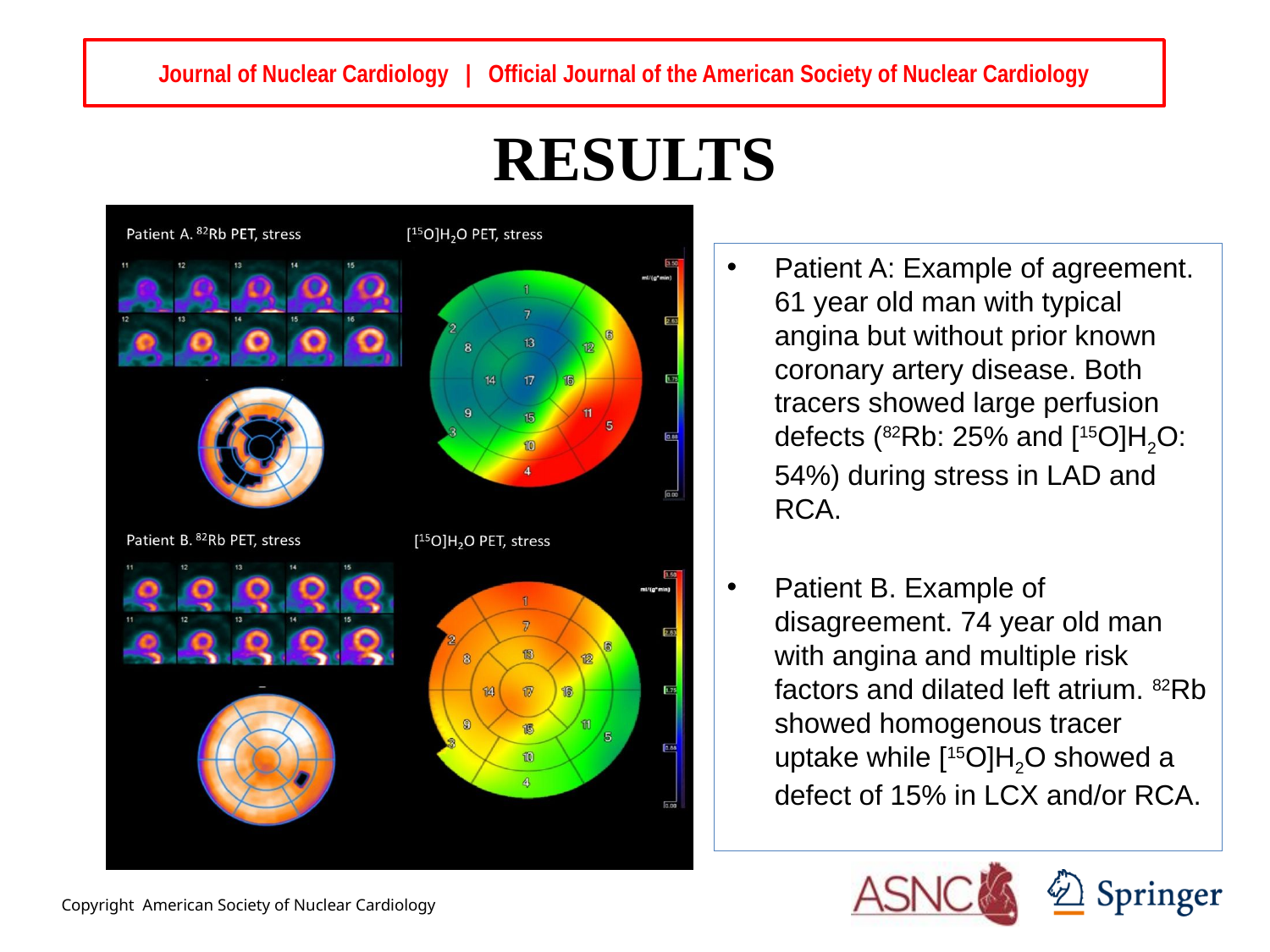

Journal of Nuclear Cardiology | Official Journal of the American Society of Nuclear Cardiology
# RESULTS
Patient A: Example of agreement. 61 year old man with typical angina but without prior known coronary artery disease. Both tracers showed large perfusion defects (82Rb: 25% and [15O]H2O: 54%) during stress in LAD and RCA.
Patient B. Example of disagreement. 74 year old man with angina and multiple risk factors and dilated left atrium. 82Rb showed homogenous tracer uptake while [15O]H2O showed a defect of 15% in LCX and/or RCA.
Copyright American Society of Nuclear Cardiology

## Slide 6
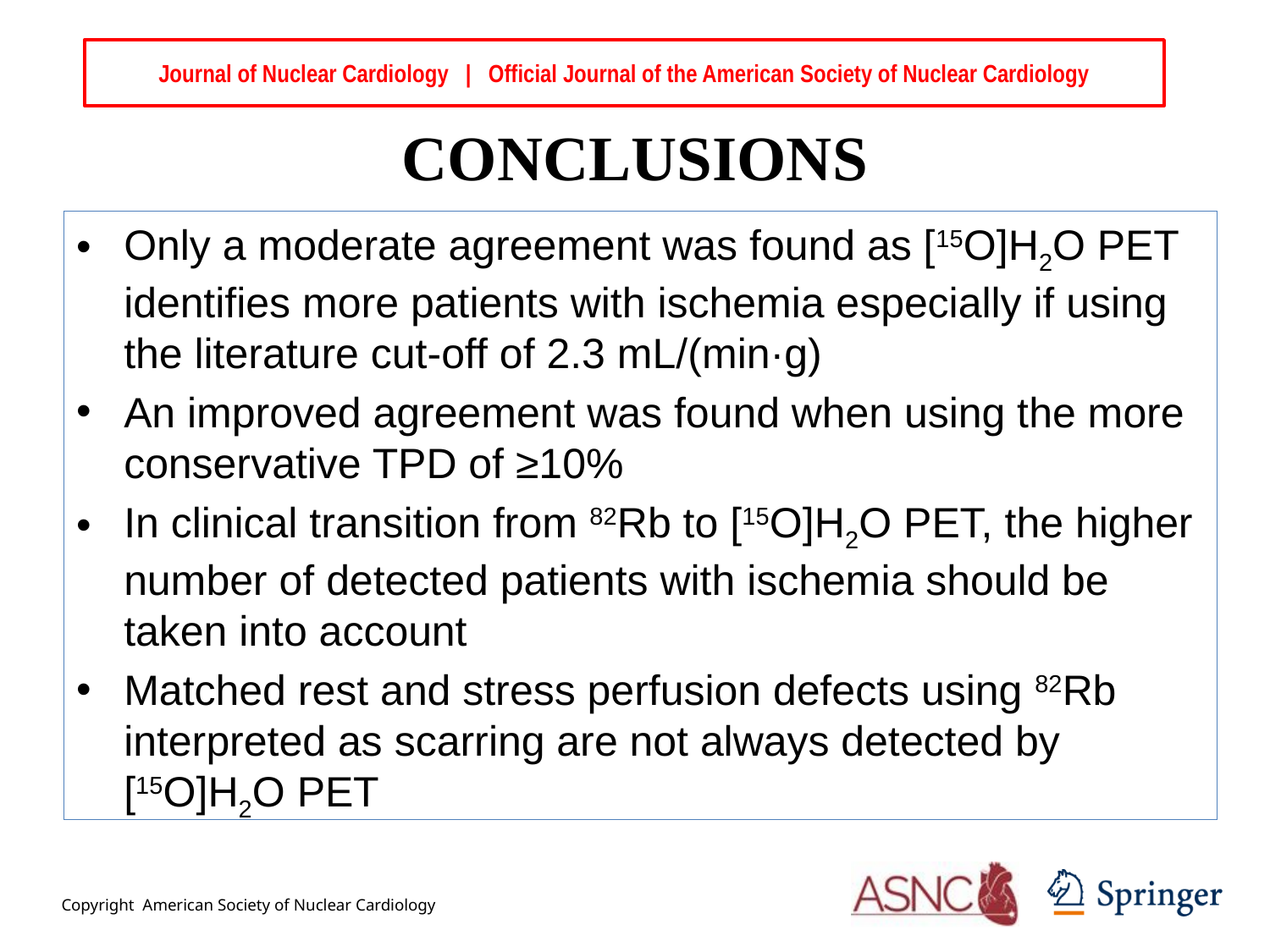

Journal of Nuclear Cardiology | Official Journal of the American Society of Nuclear Cardiology
# CONCLUSIONS
Only a moderate agreement was found as [15O]H2O PET identifies more patients with ischemia especially if using the literature cut-off of 2.3 mL/(min·g)
An improved agreement was found when using the more conservative TPD of ≥10%
In clinical transition from 82Rb to [15O]H2O PET, the higher number of detected patients with ischemia should be taken into account
Matched rest and stress perfusion defects using 82Rb interpreted as scarring are not always detected by [15O]H2O PET
Copyright American Society of Nuclear Cardiology
